# Use of Stable Isotopes to Investigate Keratin Deposition in the Claw Tips of Ducks 

**DOI:** 10.1371/journal.pone.0081026

**Published:** 2013-11-25

**Authors:** John B. Hopkins, Kyle A. Cutting, Jeffrey M. Warren

**Affiliations:** 1 Department of Ecology and Evolutionary Biology, University of California, Santa Cruz, California, United States of America; 2 Red Rock Lakes National Wildlife Refuge, U.S. Fish and Wildlife Service, Lima, Montana, United States of America; Scottish Association for Marine Science, United Kingdom

## Abstract

Stable isotopes derived from the claws of birds could be used to determine the migratory origins of birds if the time periods represented in excised sections of claws were known. We investigated new keratin growth in the claws of adult female Lesser Scaup (*Aythya affinis*) by estimating the equilibration rates of stable isotopes (*δ* ^13^C, *δ* ^15^N, and *δ* ^2^H) from the breeding grounds into 1 mm claw tips. We sampled birds on their breeding ground through time and found that it took approximately 3–3.5 months for isotope values in most claw tips to equilibrate to isotope values that reflected those present in the environment on their breeding grounds. Results from this study suggest that isotopes equilibrate slowly into claw tips of Lesser Scaup, suggesting isotopes could potentially be used to determine the wintering grounds of birds. We suggest using controlled feeding experiments or longitudinal field investigations to understand claw growth and isotopic equilibration in claw tips. Such information would be valuable in ascertaining whether claw tips can be used in future studies to identify the migratory origins of birds.

## Introduction

Stable isotope analysis has emerged as a powerful ecological tool to track the migratory patterns of birds [1]. Ecologists commonly attempt to link natural abundances of stable isotopes derived from bird tissues to isotopes associated with migratory origins (e.g., 2-7). Identifying such habitats is essential for the proper management of wetland ecosystems.

Ecologists commonly use stable isotopes derived from metabolically active tissues such as muscle and blood to identify habitats used by birds (e.g., 8-10). Such tissues undergo isotopic turnover as cells die and are replaced [11]. As a result, the isotopic composition of these tissues will change over time and reflect the diets, and therefore the habitats, utilized by birds over a variety of temporal scales [12-14]. For example, Yerkes et al. [8] found that whole blood isotope values for northern pintails (*Anas acuta*) indicate that females used a food web in the boral forest, highlighting the importance of this habitat for their breeding and staging. 

Feathers are used routinely in migration studies, but unlike muscle and blood, feathers are metabolically inert; thus, in most cases (the exception being hydrogen; [15]), the stable isotopes in feathers represent the habitats birds use during discrete time intervals when feathers were grown [12,13,16,17]. For instance, Yerkes et al. [8] found that female northern pintails that wintered or staged in coastal habitat had elevated *δ* ^13^C values compared to those that wintered or staged on inland freshwater habitat. They also found that females that rely more heavily on agricultural fields in coastal areas had elevated *δ* ^15^N values, but lower *δ* ^13^C values than conspecifics [8]. The main drawback with using feathers in such studies, however, is that molt patterns are often complex and detailed knowledge is required to link the isotope values of feathers to the isotope values of habitats occupied in the past [11]. 

Claws may be ideal for studying both contemporary and historic bird migration [18]. Unlike feathers, claws grow continuously and catalogue past dietary information into inert keratin [18]. Relating isotope values derived from excised portions of claw to discrete time periods when birds were foraging could elucidate their migratory origins. Unfortunately, the rates and patterns of claw growth are poorly understood [18,19]. For instance, Ethier et al. [18] was unable to identify a structure in claws that could undeniably offer time-series data, and Bearhop et al. [11] recorded variable growth rates by measuring claw growth in five species of palearctic passerines. These issues further exacerbate the ability of ecologists to confidently link isotopes from sections of claws to the migratory origins of birds. 

The distal segments of claws (hereafter, claw tips) have recently been used to investigate the migratory patterns of birds [11,20-24]. Unfortunately, there is significant concern about the validity of identifying discrete migratory origins (e.g., breeding grounds) using isotopes from claw tips as claws grow at different rates and their tips incorporate both old and new keratin [11,18-20]. Unlike mammalian claws, however, avian claws appear to increase in thickness distally (i.e., the portion beyond the claw containing blood vessels and nerves), suggesting continuous deposition of new keratin toward the tip [18]. Previous research on claw growth suggests that the time period represented in claw tips range from two to five months prior to sampling [11,20]; however, because deposition rates of new keratin in claw tips is unknown, the time periods represented in claw tips are also unknown. Fraser et al. [23] estimated the rate in which *δ* ^2^H was incorporated into claw tips of Golden-winged (*Vermivora chrysoptera*) and Cerulean Warblers (*Dendroica cerulean*) by sampling their claw tips on their breeding grounds through time. They found that *δ* ^2^H values decreased at a constant rate; however, isotope values for claw tips never reached isotopic equilibrium with estimated *δ* ^2^H values for their study site [23]. Therefore, the rate of isotopic change was unknown. If isotope values for claw tips of warblers decreased at a constant rate, it would be difficult to use claw tips in future studies to determine the wintering grounds of birds because some isotopes from their staging areas would have confounded the isotopic signature of the wintering grounds. 

**Figure 1:**
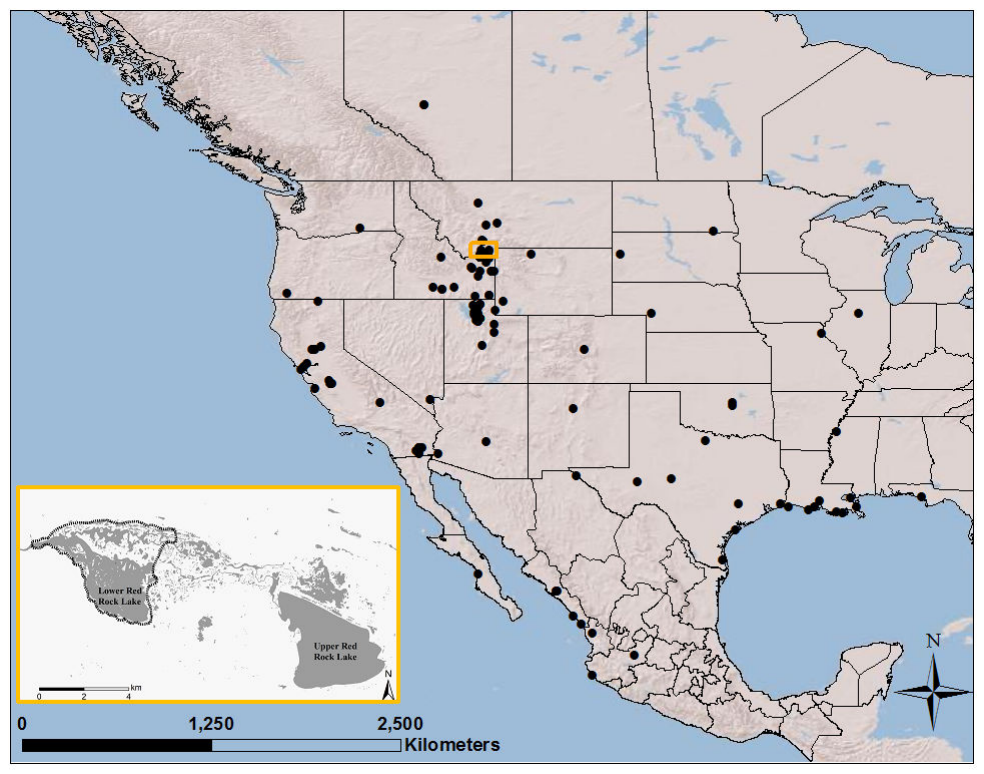
Hunter harvest recoveries of adult Lesser Scaup (*Aythya affinis*) banded at Red Rock Lakes National Wildlife Refuge (inset), Montana, U.S.A.

The goal of this study was to estimate the rate at which *δ* ^13^C, *δ* ^15^N, and *δ* ^2^H values from claw tips (distal 1 mm segment) of wild Lesser Scaup (*Aythya affinis*) that arrive on the breeding grounds equilibrate to the isotopic signature of these birds when foraging exclusively on foods consumed on the breeding grounds. Such information could be used in future studies to identify the wintering grounds of species such as Lesser Scaup sampled on their breeding grounds (Figure 1). In order to link isotopes in claw tip to the wintering grounds of individual birds, isotopes contained in claw tips must represent a past time period that is greater than the time it takes for birds to migrate to their breeding grounds. In this case, based on satellite telemetry data, Lesser Scaup migrate to Red Rock Lakes National Wildlife Refuge (Figure 1) in 32 ± 16 days (United States Geological Survey, unpublished data). Therefore, claw tips need to represent the diets of birds more than 1.5 months prior to sampling. 

We hypothesize that keratin in 1 mm claw tips would eventually contain isotopes derived exclusively from foods consumed on the breeding grounds. Our hypothesis predicts that isotope values in claw tips will equilibrate to isotope values that represent the breeding grounds. Because Lesser Scaup use a wide variety of spring staging habitats to fuel migration prior to arriving at the breeding grounds [25,26] (Figure 1), our hypothesis also predicts that isotopic variation will decline through time. Changing isotopic variation through time in claw tips, beginning when birds first arrive on the breeding grounds, suggests new keratin growth in claws. We tested these predictions by sampling claw tips of wild Lesser Scaup through time on their breeding grounds. We investigated new keratin growth in claw tips by estimating the equilibration rate of isotopes from the breeding grounds into 1 mm claw tips. We determined the approximate time period in which isotopes in 1 mm claw tips equilibrated to isotopes acquired from the breeding grounds. We used ducks as our model taxa because unlike passerines, ducks do not generally wear their claws down by foraging on the ground; such foraging tactics requires scratching that could wear claw tips and stimulate mitosis in their claws tissues, confounding the analysis [27]. 

## Materials and Methods

### Study area

Red Rock Lakes National Wildlife Refuge (hereafter, Refuge; 20,648 ha; Fig. 1) is located in the Centennial Valley in southwest Montana (44°50’N, 111°83’W). The high elevation (2,014 m above sea level) of Centennial Valley provides a narrow growing season for birds similar to the breeding areas in the north where the majority of Lesser Scaup breed (i.e., the Western Boreal Forest [28]). We sampled birds at Lower Red Rock Lake (hereafter, Lower Lake), which is a large (2,332 ha) wetland within the Refuge. Lesser Scaup usually return to Lower Lake during the first half of May when ice thaws (USFWS, unpublished data). Lower Lake is primarily open-water (<1.5 m depth during the nesting season) interspersed with islands of hardstem bulrush (*Schoenoplectus acutus*). Extensive stands of seasonally flooded Northwest Territory sedge (*Carex utriculata*) and small ponds (<2 ha) surround Lower Lake. 

### Sampling

We initiated each capture season in early May when birds returned to Lower Lake (Table 1). We captured adults (*n* = 226) via night-lighting in May–August 2008 (*n* = 109) and 2009 (*n* = 117). Proportionally more birds were captured early in the breeding season to fulfill data requirements for a concurrent demographic study. We also captured flightless ducklings and molting adults using drive-trapping [29] in August–September 2008 (172 ducklings, 3 adults) and 2009 (103 ducklings, 17 adults). 

**Table 1:** Isotopic composition ($\bar{x}$, 1 SD; *n* in parentheses) of claw tips for Lesser Scaup (*Aythya affinis*) sampled at Red Rock Lakes National Wildlife Refuge, Montana, USA, 2008 and 2009.

**Year**	**Days**	***δ*^15^N** (‰)	**δ^13^C** (‰)	**δ^2^H** (‰)
2008	0–4	+12.0 ± 2.6 (27)	-19.2 ± 4.9 (27)	
	11–25	+11.7 ± 2.6 (13)	-20.1 ± 3.3 (13)	
	51–56	+9.6 ± 1.5 (24)	-20.5 ± 1.9 (24)	
	118–119	+5.8 ± 0.2 (3)	-19.5 ± 0.4 (3)	
	Mean	+10.8 (67)	-19.5 (67)	
	1 SD	2.7	3.7	
2009	0–2	+12.6 ± 1.5 (8)	-20.0 ± 2.7 (8)	-97.3 ± 23.3 (7)
	12–16	+12.3 ± 2.5 (37)	-20.1 ± 2.7 (37)	-99.9 ± 20.9 (27)
	39–44	+10.2 ± 1.2 (30)	-20.2 ± 2.1 (30)	-125.6 ± 13.9 (36)
	69–70	+8.5 ± 0.8 (6)	-20.0 ± 0.6 (6)	-132.6 ± 9.2 (6)
	103–104	+7.5 ± 0.8 (2)	-21.5 ± 5.6 (2)	-146.8 ± 8.0 (7)
	122–124	+6.9 ± 0.8 (7)	-18.1 ± 0.5 (7)	-145.8 ± 7.6 (8)
	Mean	+10.9 (90)	-20.0 (90)	-119.7 (91)
	1 SD	2.5	2.3	23.4

Day 0 (initial capture date) corresponds to 9 May in 2008 and 11 May 2009.

We sampled birds according to protocols approved by the Animal Care and Use Committee of Montana State University (Protocol #05-07). We aged adult females (*n* = 158) as “after hatch year” (*n* = 84) or “after second year” (*n* = 74) based on eye color [30] and feather morphology. Each adult female was banded with a U.S. Geological Survey aluminum leg band and nasal marker [31]. We sampled claw tips (1 mm distal sections) from the middle and inside toe of each foot using forceps and scissors. We also sampled primary feathers (outermost feather on the right wing) from adult females in August–September 2008 (*n* = 3) and 2009 (*n* = 5); birds were sampled by collecting new feathers from the erupted quill. These resident adults were known to reside in the study area the entire breeding season based on recapture/resight data collected for a concurrent demographic study (USFWS, unpublished data). Because primary feathers for resident adult females were grown on the breeding grounds, isotope values for feathers represented the diets of birds foraging on the breeding grounds exclusively. We adjusted isotope values of primary feathers for flightless, molting, adult females to isotope values of claw tips for adult females (hereafter, adjusted feathers) using the average difference between the isotope values of feathers and claw tips of three randomly selected flightless ducklings from different crèches. We used adjusted feathers to determine when the isotope values of adult female claw tips represented the isotope values of birds that forage exclusively on the breeding grounds. This method circumvented the uncertainty associated with applying tissue-diet discrimination factors to a suite of isotope values for wetland invertebrates and plants that Lesser Scaup are known to eat. 

### Sample preparation and stable isotope analysis

We used a 2:1 chloroform-methanol rinse to wash surface oils from claws and feathers. We shredded claws from each individual into tiny pieces using a high-precision scissors. We encapsulated 1 mg of claw material into tin cups (4 x 6 mm; Costech Analytical Technologies, Inc., Valencia, CA) for carbon (*δ* ^13^C) and nitrogen (*δ* ^15^N) isotope analysis, and approximately 0.35 mg into silver cups (4 x 6 mm; Costech Analytical Technologies, Inc., Valencia, CA) for hydrogen (*δ* ^2^H) isotope analysis. The University of California, Davis Stable Isotope Facility (SIF) conducted *δ* ^13^C and *δ* ^15^N isotope analysis using a PDZ Europa ANCA-GSL elemental analyzer interfaced to a PDZ Europa 20-20 continuous flow isotope ratio mass spectrometer. SIF used conventional delta (*δ*) notation to report the relative difference of isotope ratios for samples (expressed in parts per thousand, ‰) and the international measurement standards: Vienna Peedee belemnite (VPDB) for carbon and atmospheric N_2_ (Air) for nitrogen [32,33]. SIF estimated analytical error for *δ* ^13^C and *δ* ^15^N at ±0.2 ‰ and ±0.3 ‰, respectively. 

SIF also conducted δ^2^H isotope analysis by using the comparative equilibration method described by 34; *δ* ^2^H values are equivalent to non-exchangeable feather hydrogen. SIF combusted tissues in a glassy carbon reactor at 1320°C using a Heckatech HT Oxygen Analyzer interfaced to a PDZ Europa 20-20 isotope ratio mass spectrometer. SIF used Vienna Standard Mean Ocean Water as the international measurement standard and reported stable isotope values in conventional δ notation [32,35]. Multiple repeated analyses of calibrated in-house keratin reference materials indicated a precision of ±2.1 ‰.

### Data analysis

We estimated the equilibration rate of isotopes from the breeding grounds into 1 mm claw tips. We used generalized linear models (hereafter, GLM; with normally distributed errors) with a log link to account for non-constant variance of residuals. We transformed negative *δ* ^13^C and *δ* ^2^H isotope values after adding 29 and 162 units to each value, respectively. We back-transformed intercept values from results of each model by subtracting the aforementioned values from the positive values; no back-transformation of estimated effects was necessary because the rate did not change when transforming the intercepts. 

We explored variation in the equilibration rate due to bird-age (after hatch year and after second hatch year) and year (2008 and 2009). Older females are more likely to breed [36], and breeding females have higher basal metabolic rates than non-breeding females [37]. We tested the hypothesis that older females would grow keratin faster than younger birds. This hypothesis predicts that the isotope values for older birds will equilibrate to the local study area faster than young birds. We also tested for an effect of year on equilibration rate. We aggregated isotope values for birds of different age classes and years if age and year did not have an effect on the equilibration rate of isotopes into claw tips.

We used both a parametric and non-parametric test (*t*-test and Wilcoxon rank-sum test; α = 0.05; as isotope values can be non-normally distributed) to assess annual differences in isotope values for feathers grown on the breeding grounds (to aggregate adjusted feathers between years), to compare isotope values for claw tips during capture sessions to isotope values of adjusted feathers (to confirm isotopic equilibrium between birds and the breeding grounds), and to assess whether isotope values changed during subsequent capture sessions. We conducted all statistical analyses using R 2.15.1 [38]. 

## Results

### Isotope values from claw tips

**Figure 2:**
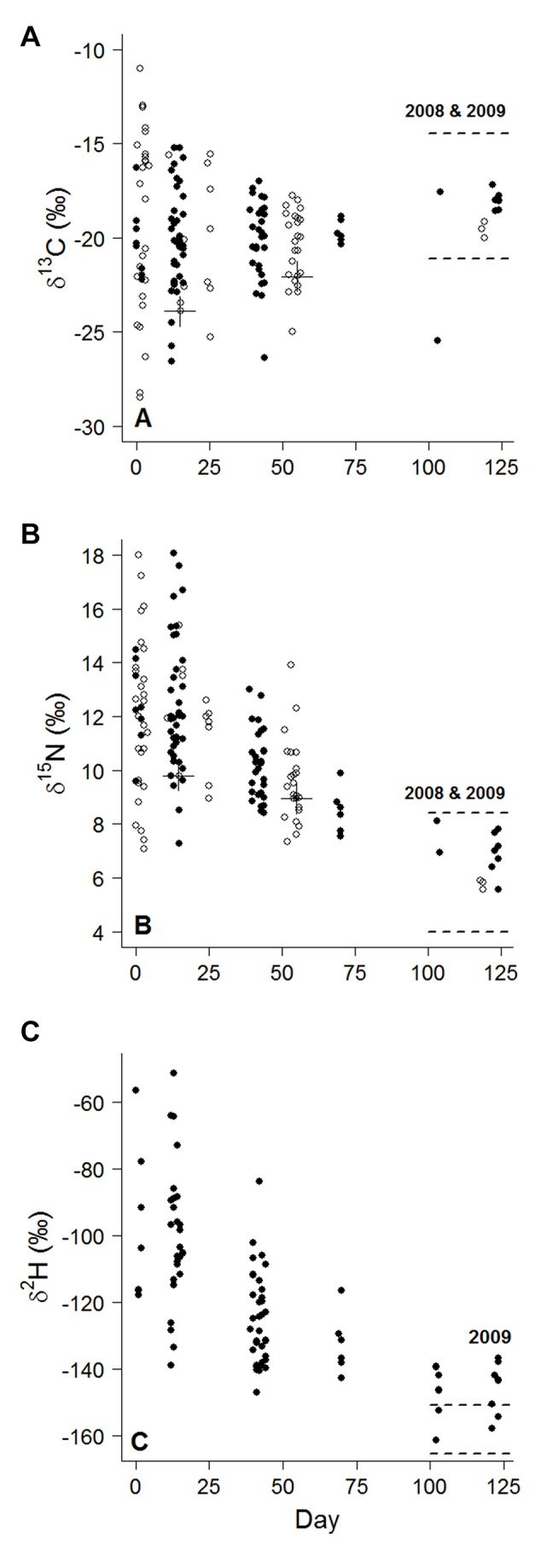
Isotopic composition of claw tips for Lesser Scaup sampled from May–September 2008 (open circles) and 2009 (closed circles) at Red Rock Lakes National Wildlife Refuge, Montana, U.S.A. Isotope values for adult female feathers (adjusted to claw tips) grown on the breeding grounds in 2008 and 2009 are denoted by dashed lines (2 SD). Day 0 (initial capture date) corresponds to 9 May in 2008 and 11 May 2009. A cross-hair denotes a recaptured individual (we sampled claw tips from the same toes).

We sampled 158 adult female birds during 2008 (*n* = 67) and 2009 (*n* = 91) (Table 1). Average (±1 SD) *δ* ^15^N values decreased from +12.1 (± 2.7) ‰ to +6.7 (± 0.9) ‰ from early May to early September 2008–2009, and average *δ* ^2^H decreased from -97.3 (± 23.30) ‰ to -145.8 (± 7.60) ‰ in 2009. The average *δ* ^13^C values did not change in both 2008 and 2009. As predicted, isotopic variation in claw tips generally decreased through time (Figure 2, Table 1). 

### Isotopic signature of the breeding grounds

Isotope values for adult female primary feathers were similar between years for *δ* ^13^C (2008: $\bar{x}=$-18.7 ± 0.7 ‰, *n* = 3; 2009: $\bar{x}=$-16.5 ± 1.5 ‰, *n* = 5; pooled: $\bar{x}=$-17.3 ± 1.7 ‰; *t*_6_ = -2.301, *P* = 0.061; *W* = 1, *P* = 0.071) and for *δ* ^15^N (2008: $\bar{x}=$+7.4 ± 0.9 ‰; 2009: $\bar{x}=$+6.9 ± 1.2 ‰; pooled: $\bar{x}=$+7.1 ± 1.1 ‰; *t*_6_ = -0.68, *P* = 0.525; *W* = 10, *P* = 0.571). We also calculated the mean *δ* ^2^H value for adult female primary feathers sampled in 2009 ($\bar{x}=$-153.4 ± 3.5 ‰). Isotope values for flightless duckling feathers were greater than isotope values for duckling claws (Table S2). We subtracted the mean difference between isotope values derived from feathers and claw tips of the same flightless ducklings grown on the breeding grounds (*δ* ^13^C = +0.5 ‰; *δ* ^15^N = +0.9; *δ* ^2^H = +5.0; Table S2) from mean values for adult female primary feathers grown on the breeding grounds. Specifically, we adjusted mean pooled *δ* ^13^C values ($\bar{x}=$-17.8 ‰) and *δ* ^15^N values ($\bar{x}=$+6.2 ‰) for 2008 and 2009, and adjusted mean *δ* ^2^H values ($\bar{x}=$-158.4 ‰) for 2009 (Figure 2). 

### Keratin deposition

We estimated the equilibration rate of isotopes from the breeding grounds into 1 mm claw tips: *δ* ^13^ *C*=*e* ^2.1936+0.0004*Day^−29 (*P*= 0.56), *δ* ^15^ *N*=*e* ^2.5447+0.0052*Day^ (*P*= <0.001), *δ* ^2^ *H*=*e* ^4.2714+0.0146*Day^−162 (*P*= <0.001). Across years, there was no difference in the rate of isotopic change throughout the breeding season for both *δ* ^13^C (GLM: *P* = 0.18) and *δ* ^15^N (GLM: *P* = 0.68). There was no relationship between the rate of isotopic change between age classes throughout the breeding season (GLM: *δ* ^13^C: *P* = 0.95, *δ* ^15^N: *P* = 0.42, and *δ* ^2^H: *P* = 0.49). Isotope values were similar during the first two sample periods for all isotopes (Figure 2; Table 1 and S1).

 The average rate of isotopic change in claw tips decreased significantly through time for *δ* ^15^N (*b* = -0.0052 ‰ ± 0.0006 (SE), *P* = <0.001) and *δ* ^2^H (*b* = -0.0146 ‰ ± 0.0019 (SE), *P* = <0.001), but not for *δ* ^13^C (*b* = 0.0004 ‰ ± 0.0008 (SE), *P* = 0.57, Figure 2). Isotope values for adjusted feathers were similar to claw tips sampled late in the breeding season (Day 103–124) for *δ* ^13^C (Figure 2A) and *δ* ^15^N (Figure 2B), indicating it takes about 3–3.5 months for 1 mm claw tip to reach *δ* ^13^C- and *δ* ^15^N-equilibrium with the study area (Table S1). *δ* ^2^H values for claw tips were significantly different than adjusted feathers late in the season (Table S1); however, some birds appeared to reach isotopic equilibrium with the breeding grounds in 3–3.5 months (Figure 2C).

## Discussion

Although our sample sizes were relatively small late in the breeding season, results from this study support the hypothesis that keratin in 1 mm claw tips will eventually contain *δ* ^13^C and *δ* ^15^N values derived exclusively from foods consumed on the breeding grounds. Unlike Fraser et al. [23], in some cases, *δ* ^2^H isotopes from the breeding grounds equilibrated into claw tips of Lesser Scaup. In general, the variation of isotope values in claw tips decreased through time for female Lesser Scaup sampled on Lower Lake, indicating new keratin growth in claws. The isotopic composition of claw tips were highly variable when birds first arrived on the breeding ground (Figure 2), suggesting birds utilized a variety of habitats prior to arrival on the Refuge. Conversely, isotope values for claw tips sampled late in the breeding season have relatively low variation and are generally similar to the isotopic values of adjusted feathers grown exclusively on the breeding grounds (Figure 2). These results are similar to controlled feeding experiments in that the isotope values of bird tissue equilibrated to the isotope values of their diets over time (e.g., [39-41]). 

Our results are consistent with results from Bearhop et al. [11] for songbirds as well as band recovery (Figure 1) and satellite data (United States Geological Survey, unpublished data) for Lesser Scaup originally marked on Lower Lake. Bearhop et al. [11] concluded that claw isotope values represented a variety of habitats utilized by songbirds during the previous winter. Band recovery data for Lesser Scaup indicated that females winter in both freshwater and marine habitats throughout the United States and Mexico in the Pacific, Central, and Mississippi Flyways (Figure 1). In addition, satellite data showed that some of these birds migrate to the Refuge using a variety of freshwater staging areas in the Pacific and Central Flyways (United States Geological Survey, unpublished data).

**Figure 3:**
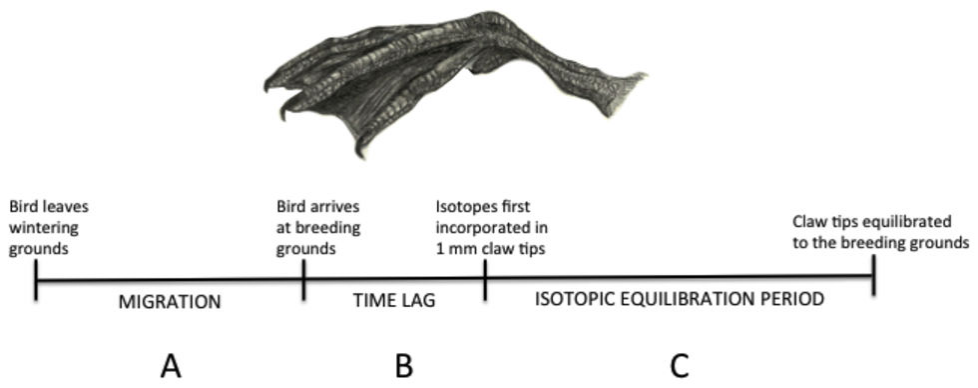
A timeline illustrating the process of isotopic equilibration in the claw tips (1 mm distal segments) of migratory birds. (A) Birds forage at staging areas along their migratory routes. (B) Isotopes from staging areas (endogenous sources) continue to assimilate into claws following arrival at the breeding grounds. Isotopes from exogenous sources are pooled in the body and are potentially being assimilated into claws, but have yet to migrate via keratin to the most proximate margin of the 1 mm claw tip. (C) Isotopes from exogenous sources begin to accumulate into claw tips. Old keratin is eroded from the distal end through time. Eventually all isotopes in the 1 mm claw tip contain isotopes acquired from the breeding grounds.

Isotopes could potentially be used to determine the wintering grounds of individual birds sampled on their breeding grounds because isotopes equilibrate into claw tips at a slow rate. We caution their use, however, until more is learned about claw growth and isotopic equilibration from both endogenous and exogenous sources. For instance, quantifying the time-lag associated with isotopes first reaching the claw tips is important to linking birds to their migratory origins. Because keratin is not a metabolic tissue, there is likely a prominent time-lag associated with isotopes from the body reaching the distal 1 mm claw tip (Figure 3). If the time-lag for new keratin growth in claw tips is greater than the time it takes for spring migration, then it is likely that claw tips fully contain isotopes obtained from their wintering grounds (Figure 3). In simple terms, if the time-lag is shorter than their spring migration, it would be difficult to link birds sampled on the breeding grounds to their wintering areas using claw tips as isotopes from their staging areas could confound the signal. 

Isotope values were similar during the first two sample periods, suggesting there may have been a time-lag (Fig. 2, Table S1). After closer examination, we detect a similar pattern as in Fraser et al. [23]. Evidence from our study suggests that 1 mm claw tips had not yet incorporated isotopes from the breeding ground in as much as 25 days after our initial capture session. We can explain this pattern as a delay in the deposition of new keratin in claw tips (Fig. 2) or that some birds arrived later on the breeding grounds. Based on results from past studies, the latter is less likely as birds typically arrive at the Refuge within a short interval (e.g., 6 radio-tagged adult female scaup arrived 7–9 May 2008; USFWS, unpublished data). Nevertheless, we suggest a controlled feeding experiment that aims to answer the question: how long does it take for new keratin, containing isotopes from the diet, to reach the 1 mm claw tip?

This study provides evidence that claws have the potential to aid ecologists in estimating the migratory origins of individual ducks (and potentially other taxa) with unknown migratory routes. In addition to controlled feeding experiments, we also recommend sampling individual birds through time, perhaps from populations with more speedy migrations. Such a longitudinal study could be used to more accurately estimate isotopic equilibration rates and time-lags of wild birds. It would also be valuable to sample birds through time on their wintering grounds to compare isotopic equilibration rates and validate estimated migratory origins. Such studies could provide ecologists the information needed to determine if future studies could use claw tips to estimate the migratory origins of their species of interest. Such studies could be conducted during relatively short intervals of time by sampling birds when they first arrive on their breeding grounds and at a relative low cost and effort compared to migration studies that use geolocators or satellite transmitters. 

As with other tissues, determining the migratory origins of individual ducks using stable isotopes from claw tips would be valuable to species conservation and management [42]. Accurately estimating the wintering grounds for individual birds using stable isotope values from claw tips of birds sampled on their breeding grounds will require: (1) identifying the wintering grounds of some birds using satellite telemetry or other migration-tracking techniques; (2) comparing isotope values of claw tips grown on the wintering grounds to isotope values of claw tips from the same birds sampled upon arrival to the breeding grounds; (3) building spatially-explicit models to estimate the wintering grounds of birds with unknown migratory origins based on isotope values from claw tips; and (4) tracking and sampling individual birds to validate estimates. Such a study would provide managers a conservation delivery tool used to determine where breeding birds winter and to identify important wintering grounds for wetland protection and enhancement. 

## Supporting Information

Table S1 **Results for t-tests and Wilcoxon rank-sum tests.**
(PDF)

Table S2 **Mean difference of isotope values for flightless duckling feathers and claw tips.**
(PDF)
