# Supplementary material for: Use of Stable Isotopes to Investigate Keratin Deposition in the Claw Tips of Ducks
Source: PLoS One. 2013 Nov 25;8(11):e81026. doi: 10.1371/journal.pone.0081026 (PMC3839900; doi:10.1371/journal.pone.0081026)
Supplement: Table S1 — Results for t-tests and Wilcoxon rank-sum tests. (PDF) [file pone.0081026.s001.pdf]

δ^{13}C-2008. Isotope values did not change through time, and were similar to adjusted feathers during the final capture session (bold).

Day	11–25	51–56	118–119	Adjusted feathers
0–4	t_{38} = 0.61, P = 0.547<br>W = 205, P = 0.407	t_{49} = 1.11, P = 0.272<br>W = 382, P = 0.281	t_{28} = 0.101, P = 0.920<br>W = 42, P = 0.948	t_{33} = -0.771, P = 0.446<br>W = 99, P = 0.743
11–25		t_{35} = 0.308, P = 0.760<br>W = 157, P = 0.988	t_{14} = -0.311, P = 0.761<br>W = 17, P = 0.800	t_{19} =-1.767, P = 0.093<br>W = 32, P = 0.161
51–56			t_{25} = -0.802, P = 0.430<br>W = 30, P = 0.699	t_{30} = -3.405, P = 0.002<br>W = 33, P = 0.006
118–119				**t_{9} = -1.672, P = 0.129**<br>**W = 3, P = 0.085**

δ^{13}C-2009. Isotope values did not generally change through time, and were similar to adjusted feathers during the final capture session (bold).

Day	12–16	39–44	69–70	103–104	122–124	Adjusted feathers
0–2	t_{43} = -0.035, P= 0.972<br>W= 144, P = 0.906	t_{36} = -0.034, P = 0.973<br>W = 114, P = 0.830	t_{12} = -0.619, P = 0.547<br>W = 15, P = 0.282	t_{8} = 0.629, P = 0.547<br>W = 9, P = 0.889	t_{13} = -2.887, P = 0.017<br>W = 7, P = 0.014	t_{14} = -2.666, P = 0.018<br>W = 9, P = 0.015
12–16		t_{65} =0.014, P = 0.989<br>W = 545, P = 0.900	t_{41} = -0.419, P = 0.677<br>W = 85, P = 0.353	t_{37} = 0.659, P = 0.514<br>W = 43, P = 0.702	t_{42} = -2.042, P = 0.048<br>W = 56, P = 0.018	t_{43} = -2.337, P = 0.024<br>W = 61, P = 0.010
39–44			t_{34} =-0.563, P = 0.577<br>W = 80, P = 0.671	t_{30} = 0.818, P = 0.419<br>W = 31, P = 0.938	t_{35} = -2.708, P = 0.010<br>W = 34, P = 0.006	t_{36} = -2.992, P = 0.005<br>W = 45, P = 0.007
69–70				t_{6} = 0.965, P = 0.372<br>W = 6, P = 0.999	t_{11} = -5.65, P = <0.001<br>W = 0, P = 0.001	t_{12} = -2.651, P = 0.021<br>W = 5, P = 0.013
103–104					t_{7} =-2.047, P = 0.081<br>W = 6, P = 0.889	**t_{8} = -1.886, P = 0.091**<br>**W = 5, P = 0.533**
122–124						**t_{13} = -0.344, P = 0.736**<br>**W = 31, P = 0.779**

δ^{15}N-2008. Isotope values were similar during the first capture session (italics); different during subsequent capture sessions; and similar to adjusted feathers during the final capture session (bold).

Day	11–25	51–56	118–119	Adjusted feathers
0–4	*t_{38} = -0.317, P = 0.753*<br>*W = 164, P = 0.754*	t_{49} = 3.55, P = <0.001<br>W = 484, P = 0.003	t_{28} = 3.54, P = 0.001<br>W = 81, P = <0.001	t_{33} = 5.323, P = <0.001<br>W = 215, P = <0.001
11–25		t_{35} = 4.38, P = <0.001<br>W = 263, P = 0<001	t_{14} = 4.943, P = <0.001<br>W = 39, P = 0.0.004	t_{19} = 7.190, P = <0.001<br>W = 104, P = <0.001
51–56			t_{25} = 4.25, P =<0.001<br>W = 72, P = 0.034	t_{30} = 5.774, P = <0.001<br>W = 191, P = <0.001
118–119				**t_9 = -0.645, P = 0.535**<br>**W = 6, P = 0.279**

δ^{15}N-2009. Isotope values were similar during the first capture session (italics); different during subsequent capture sessions; and similar to adjusted feathers during the final capture sessions (bold).

Day	12–16	39–44	69–70	103–104	122–124	Adjusted feathers
0–2	*t_{43} = 0.256, P = 0.799*<br>*W = 172, P = 0.493*	t_{36} =4.284, P = <0.001<br>W = 208, P = 0.002	t_{12} = 5.466, P = <0.001<br>W = 47, P = 0.001	t_8 = 4.079, P = 0.004<br>W = 16, P = 0.044	t_{13} = 8.319, P = <0.001<br>W = 56, P = <0.001	t_{14} = 9.147, P = <0.001<br>W = 64, P = <0.001
12–16		t_{65} =4.17, P = <0.001<br>W= 871, P =<0.001	t_{41} = 3.719, P = <0.001<br>W = 210, P = <0.001	t_{37} = 2.718, P = 0.009<br>W = 73, P = 0.005	t_{42} = 5.732, P = <0.001<br>W = 257, P = <0.001	t_{43} = 6.898, P = <0.001<br>W = 295, P = <0.001
39–44			t_{34} = 3.189, P = 0.003<br>W = 162, P = 0.001	t_{30} = 2.969, P = 0.006<br>W = 60, P = 0.004	t_{35} = 6.663, P = <0.001<br>W = 210, P = 0.<0.001	t_{36} = 8.311, P = <0.001<br>W = 240, P = <0.001
69–70				t_6 = 1.41, P = 0.208<br>W = 10, P = 0.289	t_{11} = 3.540, P = 0.004<br>W = 39, P = 0.008	t_{12} = 4.304, P = 0.001<br>W = 48, P = <0.001
103–104					**t_7 = 0.987, P = 0.357**<br>**W = 10, P = 0.500**	**t_8 = 1.603, P = 0.148**<br>**W = 15, P = 0.089**
122–124						**t_{13} = 1.473, P = 0.165**<br>**W = 41, P = 0.148**

δ^2H -2009. Isotope values were similar during the first capture session (italics), and were different during subsequent capture sessions and adjusted feathers.

Day	12–16	39–44	69–70	103–104	122–124	Adjusted feathers
0–2	*t_{32} = 0.289, P = 0.774*<br>*W = 92, P = 0.915*	t_{41} = 4.405, *P* = <0.001<br>*W* = 222, *P* = <0.001	t_{11} = 3.474, *P* = 0.005<br>*W* = 41, *P* = 0.002	t_{12} = 5.321, *P* = <0.001<br>*W* = 49, *P* = <0.001	t_{13} = 5.585, *P* = <0.001<br>*W* = 56, *P* = <0.001	t_{10} = 5.744, *P* = <0.001<br>*W* = 35, *P* = 0.002
12–16		t_{61} = 5.89, *P* = <0.001<br>*W* = 836, *P* = <0.001	t_{31} = 3.72, *P* = <0.001<br>*W* = 152, *P* = <0.001	t_{31} = 3.72, *P* = <0.001<br>*W* = 152, *P* = <0.001	t_{32} = 5.786, *P* = <0.001<br>*W* = 189, *P* = <0.001	t_{30} = 6.178, *P* = <0.001<br>*W* = 135, *P* = <0.001
39–44			t_{40} = 1.180, *P* = 0.245<br>*W* = 136, *P* = 0.323	t_{41} = 3.892, *P* = <0.001<br>*W* = 236, *P* = <0.001	t_{42} = 3.959, *P* = <0.001<br>*W* = 266, *P* = <0.001	t_{39} = 5.213, *P* = <0.001<br>*W* = 180, *P* = <0.001
69–70				t_{11} = 2.989, *P* = 0.012<br>*W* = 39, *P* = 0.008	t_{12} = 2.943, *P* = 0.012<br>*W* = 43, *P* = 0.017	t_9 = 5.912, *P* = <0.001<br>*W* = 30, *P* = 0.004
103–104					t_{13} = -0.247, *P* = 0.809<br>*W* = 26, *P* = 0.867	t_{10} = 3.019, *P* = 0.013<br>*W* = 31, *P* = 0.030
122–124						t_{11} = 3.432, *P* = 0.006<br>*W* = 37, *P* = 0.011
