# Supplementary material for: Use of Stable Isotopes to Investigate Keratin Deposition in the Claw Tips of Ducks
Source: PLoS One. 2013 Nov 25;8(11):e81026. doi: 10.1371/journal.pone.0081026 (PMC3839900; doi:10.1371/journal.pone.0081026)
Supplement: Table S2 — Mean difference of isotope values for flightless duckling feathers and claw tips. (PDF) [file pone.0081026.s002.pdf]

Table S2. Mean difference of isotope values for flightless duckling feathers and claw tips.

Band #	Tissue	$\delta^{13}C$	$\delta^{15}N$	$\delta^{2}H$
89257	Feather	-18.3	+4.7	-148.5
	Claw tip	-18.9	+3.6	-157.1
	Difference	0.6	1.2	8.6
59258	Feather	-18.3	+4.7	-157.2
	Claw tip	-19.3	+4.2	-161.0
	Difference	1.0	0.5	3.8
89256	Feather	-19.1	+5.0	-150.9
	Claw tip	-19.0	+4.0	-153.3
	Difference	-0.1	1.0	2.4
	Mean Difference	0.5	0.9	5.0
